# North American historical monthly spatial climate dataset, 1901–2016

**DOI:** 10.1038/s41597-020-00737-2

**Published:** 2020-11-23

**Authors:** Heather MacDonald, Daniel W. McKenney, Pia Papadopol, Kevin Lawrence, John Pedlar, Michael F. Hutchinson

**Affiliations:** 1 Natural Resources Canada – Canadian Forest Service, Great Lakes Forestry Centre, Research Scientist, P6A 2E5 1219 Queen Street East, Sault Ste. Marie, Ontario, Canada; 2 Natural Resources Canada – Canadian Forest Service, Great Lakes Forestry Centre, Chief, Landscape Analysis and Applications, P6A 2E5 1219 Queen Street East, Sault Ste. Marie, Ontario, Canada; 3 Natural Resources Canada – Canadian Forest Service, Great Lakes Forestry Centre, Visiting Scientist, P6A 2E5 1219 Queen Street East, Sault Ste. Marie, Ontario, Canada; 4 Natural Resources Canada – Canadian Forest Service, Great Lakes Forestry Centre, Geographical Information Systems Analyst, P6A 2E5 1219 Queen Street East, Sault Ste. Marie, Ontario, Canada; 5 Natural Resources Canada – Canadian Forest Service, Great Lakes Forestry Centre, Forest Resource Biologist, P6A 2E5 1219 Queen Street East, Sault Ste. Marie, Ontario, Canada; 6 Fenner School of Environment and Society, Australian National University, Canberra, Australia

**Keywords:** Atmospheric science, Hydrology

## Abstract

We present historical monthly spatial models of temperature and precipitation generated from the North American dataset version “j” from the National Oceanic and Atmospheric Administration’s (NOAA’s) National Centres for Environmental Information (NCEI). Monthly values of minimum/maximum temperature and precipitation for 1901–2016 were modelled for continental United States and Canada. Compared to similar spatial models published in 2006 by Natural Resources Canada (NRCAN), the current models show less error. The Root Generalized Cross Validation (RTGCV), a measure of the predictive error of the surfaces akin to a spatially averaged standard predictive error estimate, averaged 0.94 °C for maximum temperature models, 1.3 °C for minimum temperature and 25.2% for total precipitation. Mean prediction errors for the temperature variables were less than 0.01 °C, using all stations. In comparison, precipitation models showed a dry bias (compared to recorded values) of 0.5 mm or 0.7% of the surface mean. Mean absolute predictive errors for all stations were 0.7 °C for maximum temperature, 1.02 °C for minimum temperature, and 13.3 mm (19.3% of the surface mean) for monthly precipitation.

## Abstract (summary)

**Table:** 

Measurement(s)	temperature • hydrological precipitation process
Technology Type(s)	computational modeling technique • digital curation
Factor Type(s)	monthly values of minimum/maximum temperature and precipitation
Sample Characteristic - Environment	climate system
Sample Characteristic - Location	contiguous United States of America • Canada

Machine-accessible metadata file describing the reported data: 10.6084/m9.figshare.13110422

## Background & Summary

Climate data in North America are collected through a system of weather stations distributed unevenly throughout the continent. Practical uses often call for climate data far away from the meteorological stations. This need is filled by “spatially modelled” climate data that estimate climate values from historical weather observation networks. These spatial models are important in fields such as hydrology, horticulture, power generation and agriculture, among others. In particular, the monthly time step is highly useful. While for some applications, the daily timescale is preferable, monthly data sets typically have a lower prediction error, and can provide a useful alternative to researchers for whom daily data can quickly become computationally or otherwise unmanageable.

In this paper, we present updated historical monthly models of mean maximum and minimum temperature and total precipitation from 1901 to 2016, available at 10.26050/WDCC/CCH_3876085^1^. The North American dataset (Northam version “j”)^2^ was downloaded from the National Oceanic and Atmospheric Administration’s (NOAA’s) National Centres for Environmental Information. These data are publicly distributed at https://data.nodc.noaa.gov/cgi-bin/iso?id = gov.noaa.ncdc:C00949. We utilized ANUSPLIN^3^ to produce thin plate smoothing spline models of North American historical monthly mean values of daily minimum/maximum temperature and total precipitation from 1901 to 2016. McKenney *et al*.^4^ published similar models of these variables and ancillary bio-climate indices covering Canada and the United States (see also McKenney *et al*.^5^). We compare error estimates and surface diagnostics between the two sets of analyses, and report on features associated with the new models.

The current models are based on a greater number of stations compared to North American monthly historical spatial models published in 2006^4^. For the 2006 models, relatively few stations were available in the first half of the 20th century; 1,221 stations were available from the U.S. Historical Climatology Network^6^, with an additional 46 stations for Alaska, and between 81 and 1742 stations from the Meteorological Service of Canada (from 1901 to 1993). The number of U.S. stations available in 2006 increased from over 5,000 stations between 1951 to over 7600 stations between 1971 and 2000.

The RTGCV, a measure of the predictive error akin to a spatially averaged standard predictive error estimate, averaged 0.94 °C for maximum temperature, 1.33 °C for minimum temperature, and 25.2% of the surface mean for precipitation from 1901 to 2016. RTGCVs for the current models were lower than those from 2006^4^ for maximum temperature (1.03 °C) and precipitation (>30%), and similar for minimum temperature (1.3 °C). MAEs (all stations) averaged 0.71 °C (minimum temperature), 1.02 °C (minimum temperature), and 13.31 mm, or 19.3% of the surface mean (precipitation). While differences exist in methodologies between the 2006 and current models, MAEs for precipitation were typically more than 30% of observed precipitation for the 2006 models, considerably larger than those presented in this study (under 20%).

Much of the improvement in the current models is due to efforts by NOAA and other agencies to rescue and restore historical temperature and precipitation records, as well as improved quality control processes. Northam “j” records, which have been subjected to a homogenization process, identified observations failing one or more quality control tests. The models presented in this paper also benefited from more systematic anomaly detection using studentized residuals. Despite these improvements, there are regional variations in model predictive accuracy, with coastal (Pacific and Atlantic) stations having the highest levels of predictive error for precipitation, particularly in the winter.

## Methods

### Source data

We used the North American Dataset (“Northam”) from the National Oceanic and Atmospheric Administration’s (NOAA’s) National Centres for Environmental Information^2^. The Northam data are generated from the Global Historical Climate Network-Monthly (GHCN-M) dataset^7^. Northam has been the calibration dataset for the U.S. Historical Climate Network (USHCN) since version 2.

We downloaded version “j” of Northam. Northam version “j” values were subjected to a pairwise homogenization algorithm described in Menne and Williams^8^. The nature and quality of these homogenized data have been analysed in numerous published articles^9–11^. GHCN-M quality control procedures are documented in Lawrimore *et al*.^7^. Observations flagged by NOAA through this process as having one or more quality control issues^7,12^ were dropped from our analysis^13^. A description of the GHCN quality data flags (“QFLAG”s) is provided at https://www1.ncdc.noaa.gov/pub/data/ghcn/daily/readme.txt.

**Fig. 1:**
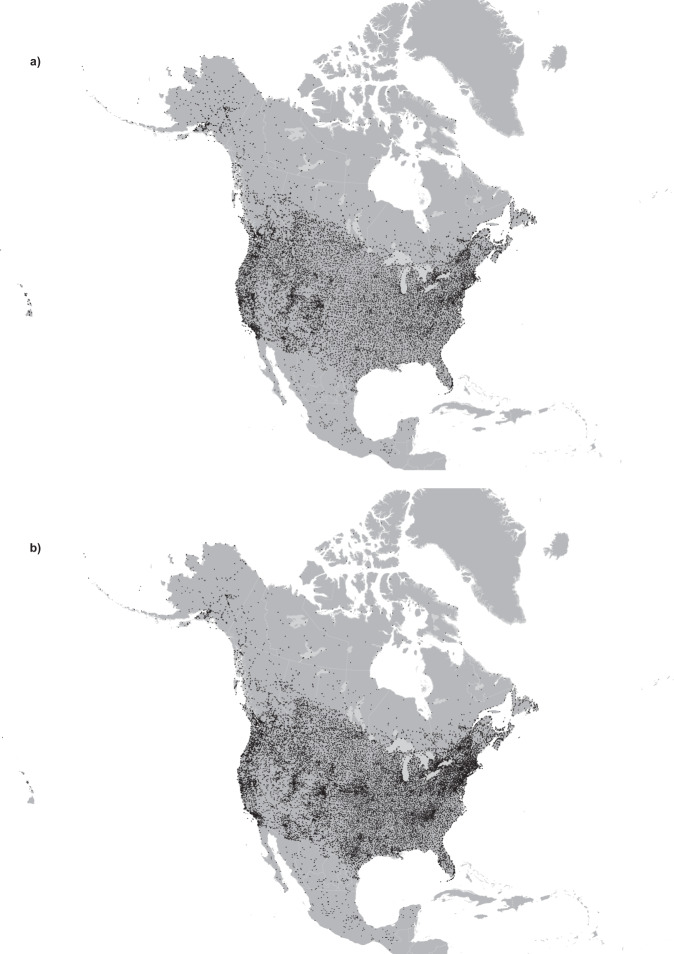
Map of Northam “j” temperature (**a**) and precipitation (**b**) stations used for the current models (Mexican stations were not covered by the digital elevation model).

**Fig. 2:**
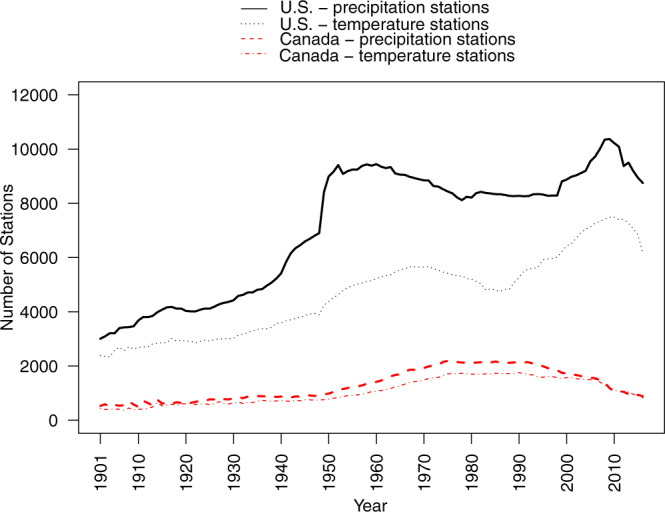
Number of temperature and precipitation stations by year in Canada and in the United States.

Figure 1 is a map of temperature and precipitation stations used for the analysis (see also^13^). Figure 2 illustrates the number of stations by variable by country by year. The number of Canadian precipitation station records increased from 528 in 1901 to over 2,000 stations from 1971 to 1993. The number of Canadian station records then declined to under 1,000 stations by 2012. In contrast, U.S. records increased from just over 3,000 in 1901 to over 8,000 from 1950 onwards.

### Spatial modeling

We used thin plate smoothing spline algorithms as implemented in ANUSPLIN^3^. ANUSPLIN is a suite of FORTRAN programs under development for more than 25 years for applying thin plate spline data smoothing techniques to multi-variate data. ANUSPLIN has been used by researchers around the world^14–16^. Here we only provide a brief description of thin plate splines. Readers are directed to Wahba^17^ for a more detailed description of thin plate splines. Hutchinson^18^ gives a general model for a thin plate spline function *f* fitted to n data values z_i_ at position x_i_:

$${{\rm{z}}}_{{\rm{i}}}={\rm{f}}\left({{\rm{x}}}_{{\rm{i}}}\right)+{{\rm{\varepsilon }}}_{{\rm{i}}}\left({\rm{i}}=1\ldots {\rm{n}}\right)$$

in which the x_i_ refer to the independent variables, in this case, longitude, latitude and elevation multiplied by a factor of 100. Multiplying elevation by a factor of 100, which reflects the relative horizontal and vertical scales of atmospheric dynamics^19^, has been shown to improve predictive performance as demonstrated by Hutchinson^20^ and Johnson *et al*.^15^. The ε_i_ are mean random errors that represent both measurement error as well as model deficiencies, reflecting localized effects below the resolution of the data network such as cold air drainage.

Precipitation values were subjected to a square root transformation prior to surface fitting. The square root transformation reduces skewness of the precipitation variable^19^, making the application of a fixed level of smoothing more consistent between small and large precipitation values across the data network. Tait *et al*.^21^ have confirmed that the square root transformation can yield a significant reduction in daily precipitation interpolation error. This transformation also makes the detection of data errors more consistent between small and large precipitation values.

The model solution was obtained by minimizing the generalised cross validation (GCV)^4^. The GCV is calculated by implicitly removing each data point and summing a suitably weighted square of the difference of each omitted data point from a surface fitted to all remaining data points^17^.

For large datasets, ANUSPLIN uses a sample of stations, called knots, to construct the thin plate smoothing spline surfaces. The use of knots reduces the computational complexity while still making use of every data point to calculate the fitted surface^3^. In this case, approximately 40% of data points were selected as knots.

In the course of modeling, we reviewed the signal, which is a diagnostic metric produced by ANUSPLIN. The signal statistic ranges between zero and the number of knots. Hutchinson and Gessler^22^ suggest that the signal should generally be no greater than about half the number of selected data points. Models with a good signal provide a balance between data smoothing and exact interpolation, while models with signals approaching the number of data points result in a rougher surface that approaches an exact interpolation of the source data. Exact interpolations reflect a model that is less robust, particularly in regions with few stations.

During the course of model development, root generalized cross validation (RTGCV) values in the log files output by ANUSPLIN’s SPLINE program were also reviewed. The RTGCV, the square root of the GCV described above, is an estimate of predictive standard error. Of note, the RTGCV is a conservative measure of standard error, because it includes data error as estimated by the ANUSPLIN program^3^.

ANUSPLIN flags data values that exceed a user-set threshold for studentized residuals, greatly improving the analyst’s ability to systematically detect anomalous recorded values and potential errors. For the current analysis, we examined flagged cases with a studentized residual of greater than 3.71. The probability of exceedance corresponds to a student’s t distribution^23^. Following a comparison of flagged values against observations from neighbouring stations, we removed cases with studentized residuals exceeding 3.71; 0.05% of stations for minimum temperature, 0.15% for maximum temperature, and 0.17% for precipitation^13^. While these observations were not used to develop the gridded estimates, they were still retained to assess the quality of model predictions.

The CV estimates taken from SPLINE’s output point cross validation file were used to calculate the mean error (ME) statistics presented in this study, calculated as the cross-validation estimate less recorded station values. In addition, the mean absolute error (MAE) was calculated for a set of 160 stations^13^ for January, April, July, and October at 5-year intervals from 1905 to 2015. This set of stations was selected to reflect a representative and high quality sample compared to using all stations, which would under-represent northern stations. Please see https://www1.ncdc.noaa.gov/pub/data/ghcn/daily/ghcnd-stations.txt for a list of all GHCN stations (including those outside of North America) and their metadata.

As a final summary, MEs and MAEs were calculated for all stations by season (winter: December, January, and February; spring: March, April, and May; summer: June, July, and August; and autumn: September, October, and November). SAS software, Version 9.4 of the SAS System for Windows, was used to calculate differences between the predicted and recorded values, as well as to conduct correlation (PROC CORR) analyses on MEs and MAEs for the set of 160 test stations. Error maps were created in ArcGIS^24^.

## Data Records

Monthly grids of mean maximum/minimum temperature, and total precipitation were generated between 1901 and 2016 using a 60 arc-second (approximately 2 km) Digital Elevation Model covering the continental US and Canada are archived at the World Data Center for Climate (WDCC) at DKRZ^1^. Post- 2016 grids are regularly published to the same DOI, and may also be obtained by contacting the corresponding author.

These monthly historical spatial models cover the geographic area from −168° to −52° longitude, and from 25° to 85° latitude from 1950 to 2016. Because of the small number of northern weather stations from 1901 to 1949, monthly historical models over this time cover a reduced area (−168° to −52° longitude, and from 25° to 60° latitude).

## Technical Validation

Table 1 summarizes RTGCV statistics output from ANUSPLIN’s log files for monthly mean maximum/minimum temperature and total precipitation for current models as well as for those published in 2006^4^. RTGCVs for the current models are smaller for maximum temperature (0.94 °C) and precipitation (25.2% of the surface mean) compared to the 2006 models (1.03 °C and >30% respectively). The RTGCV for minimum temperature averaged 1.3 °C, similar to the 2006 models.

**Table 1:** Average RT GCV for monthly historical models by month based on Northam “j” (1901–2016) compared to 2006 models (McKenney *et al*., 2006; 1901–2000).

Month	Northam “j” Models	2006 Models	Northam “j” Models	2006 Models	Northam “j” Models	2006 Models
	Maximum temperature (°C)		Minimum temperature (°C)		Precipitation (% of surface mean)	
January	0.96	1.01	1.40	1.41	25.7%	29.7%
February	0.93	1.00	1.37	1.40	26.3%	30.5%
March	0.91	0.99	1.25	1.24	24.9%	29.5%
April	0.92	1.00	1.19	1.15	24.8%	29.3%
May	0.95	1.04	1.22	1.17	25.0%	30.2%
June	0.99	1.11	1.29	1.21	26.9%	33.1%
July	1.03	1.14	1.36	1.27	29.3%	36.8%
August	1.00	1.12	1.40	1.32	29.7%	37.0%
September	0.94	1.05	1.43	1.37	27.9%	33.6%
October	0.88	0.96	1.40	1.40	26.1%	31.3%
November	0.89	0.93	1.34	1.34	24.7%	29.1%
December	0.93	0.97	1.36	1.36	24.9%	29.2%
Total	0.94	1.03	1.33	1.30	25.2%	31.6%

The average RTGCV was 0.94 °C for maximum temperature, lower than that for minimum temperature (1.33 °C). The larger errors for minimum temperature are consistent with previous work and reflect shorter length scales and larger observational/representativeness errors for this variable compared to maximum temperatures. As noted by Hutchinson *et al*.^18^, maximum temperature patterns are strongly controlled by ground elevation intersecting linear atmospheric lapse rates but minimum temperature patterns are controlled by additional processes, including cold air drainage which inverts local lapse rates, particularly in winter months^25,26^. Errors for maximum temperature are slightly larger in mid-summer, reflecting the greater variability of the higher temperature values^19^.

RTGCVs for minimum temperature ranged from 1.19 °C in April to 1.4 °C in October. RTGCVs for maximum temperature were largest in July (1.03 °C) and lowest in October (0.88 °C). For precipitation, the RTGCVs were largest in the summer (July and August), similar to the pattern reported by McKenney *et al*.^4^ This pattern reflects greater spatial complexity of convective rainfall compared to frontal precipitation occurring in winter months. Consistent with Hutchinson *et al*.^19^, we found lowest predictive errors in autumn for maximum temperature and precipitation, “consistent with the large well-organized synoptic systems that prevail in this season” (p. 725).

The signal to number of knots ratio for the current models ranged between 47.0% and 64.7% for minimum temperature, and 39.5% to 60.0% for maximum temperature. For total monthly precipitation, the signal to knots ratio showed a wider range from 26.4% to 62.9%, but no problematic surfaces, as defined by Hutchinson & Gessler^13,22^.

**Fig. 3:**
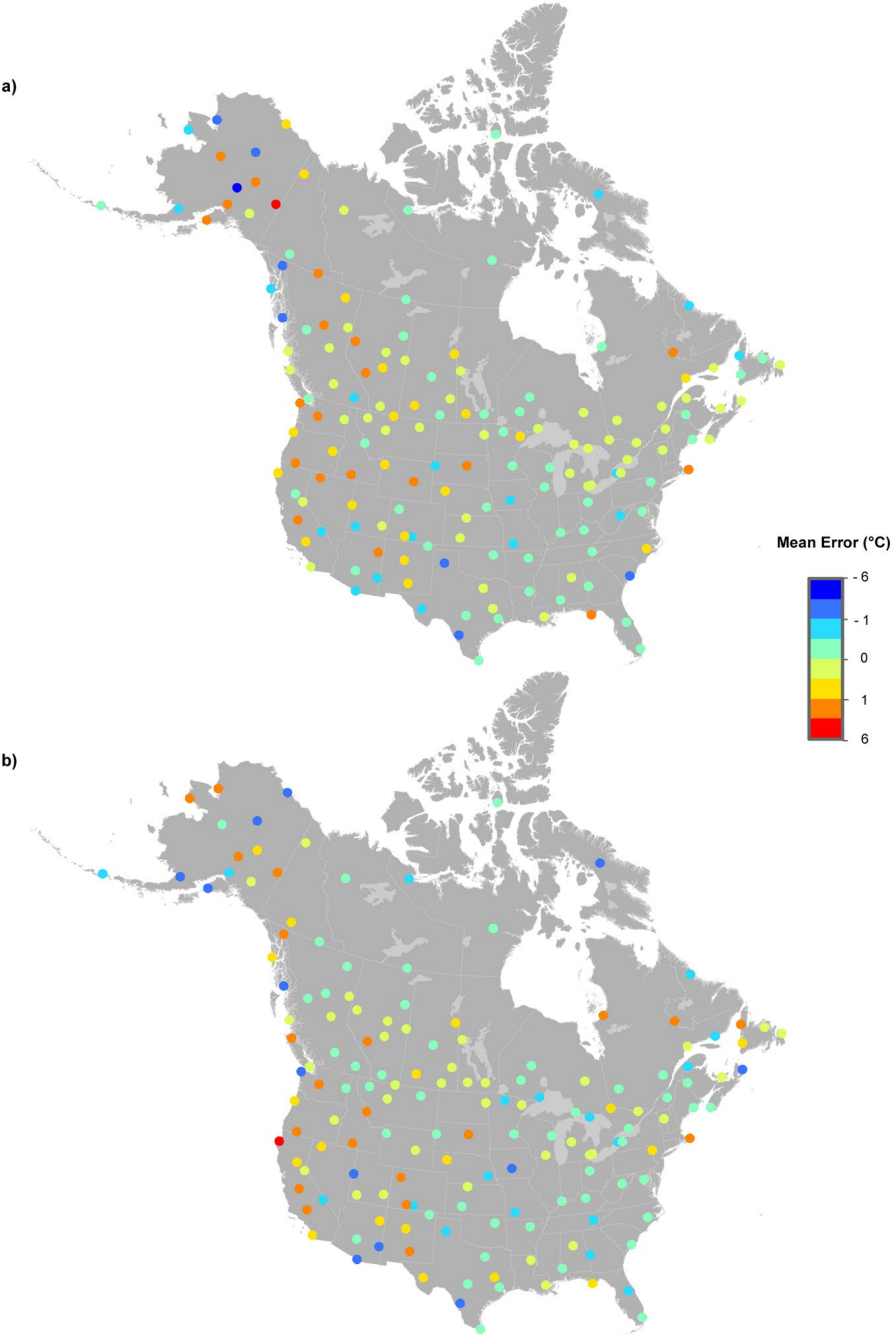
Spatial variation in errors at 160 weather stations for maximum temperature (°C). MEs were calculated by subtracting actual recorded values from estimates from 1950 to 2015 (every five years) for (**a**) January and (**b**) July.

**Fig. 4:**
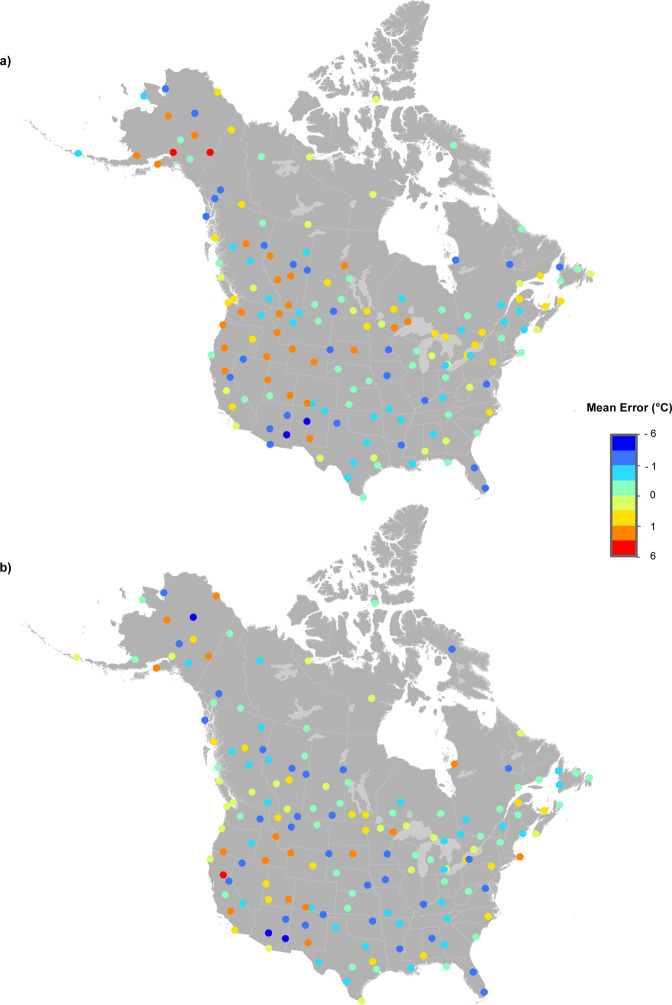
Spatial variation in errors at 160 weather stations for minimum temperature (°C). MEs were calculated by subtracting actual recorded values from estimates from 1950 to 2015 (every five years) for (**a**) January and (**b**) July.

**Fig. 5:**
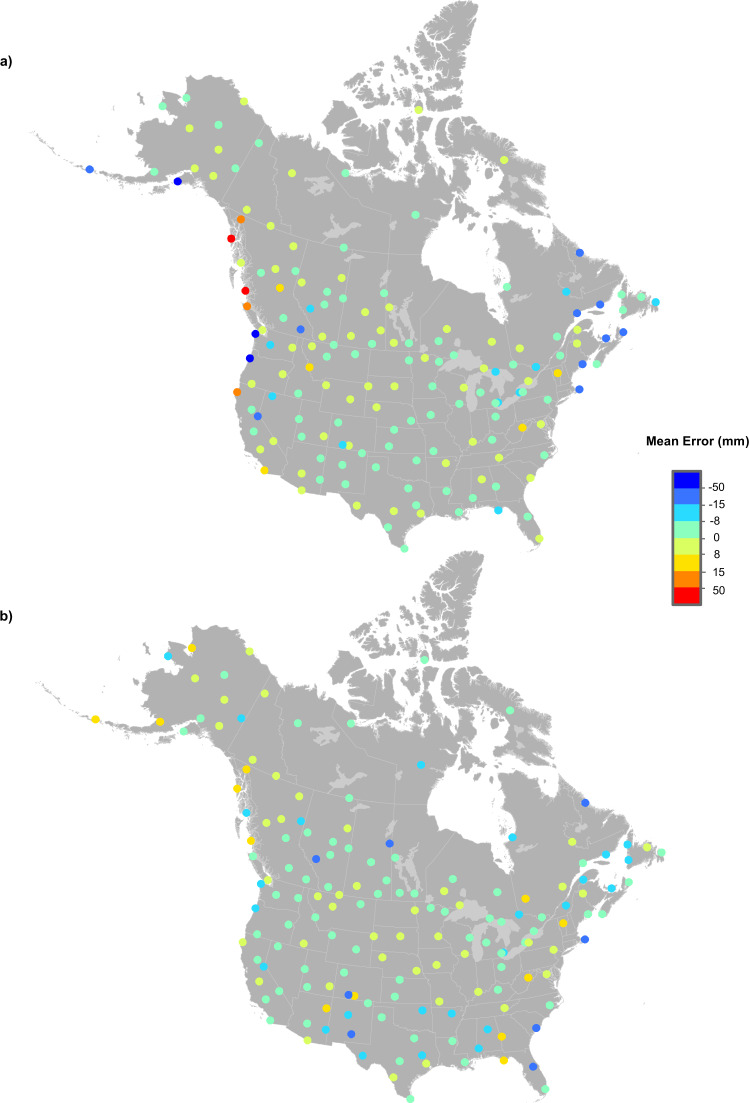
Spatial variation in errors at 160 weather stations for total precipitation (mm). MEs were calculated by subtracting actual recorded values from estimates from 1950 to 2015 (every five years) for (**a**) January and (**b**) July.

Figures 3 to 5 illustrate the MEs (Estimated less Recorded) and MAEs for January, April, July and October for 160 test stations every five years from 1905 to 2015. Maximum temperature model errors were typically between –3.0° and 3.0 °C (Fig. 3). Minimum temperature predictive errors were larger than those for maximum temperature (Fig. 4). Model errors for total monthly precipitation (Fig. 5) were largest at the coasts in January. July precipitation errors were substantially smaller, falling mostly between ±10 mm as compared to ±50 mm for January precipitation errors. Larger model errors were evident along the Pacific and Atlantic coasts for January precipitation, linked to heavy and highly variable winter precipitation events.

Across the entire dataset, the MAE for maximum temperature was 0.71 °C, compared to 1.02 °C for minimum temperature. MAEs were largest in summer for maximum temperature, and in winter for minimum temperature as shown in Table 2. MAEs for precipitation were largest in the summer (21% of the surface mean) compared to spring (17.9%). The spatial models exhibited a negative (or dry) bias relative to recorded values for precipitation of 0.5 mm or 0.7% of the surface mean. In comparison, MEs for temperature variables were less than 0.01 °C.

**Table 2:** Average cross-validation MAEs and MEs for the entire dataset by season and overall.

Season	Maximum temperature			Minimum temperature			Precipitation		
	Number of Observations	ME (°C)	MAE (°C)	Number of Observations	ME (°C)	MAE (°C)	Number of Observations	ME (mm)	MAE in mm (%)
Spring	5,606,375	0.007	0.71	5,548,625	0.0002	0.94	7,562,559	−0.44 (−0.6%)	12.57 (17.9%)
Summer	5,610,784	0.005	0.75	5,549,952	−0.003	1.01	7,572,416	−0.51 (−0.7%)	15.67 (21.0%)
Autumn	5,610,312	0.005	0.68	5,554,608	−0.004	1.05	7,570,562	−0.54 (−0.8%)	12.85 (19.3%)
Winter	5,602,647	0.002	0.74	5,548,834	−0.003	1.07	7,556,832	−0.52 (−0.8%)	12.04 (18.6%)
Total	22,430,118	0.005	0.72	22,202,019	−0.002	1.02	30,262,369	−0.50 (−0.7%)	13.31 (19.3%)

Average MAEs/MEs were calculated by year and then averaged from 1901–2016. Total number of observations represents the sum of observations in each season or year (total).

**Fig. 6:**
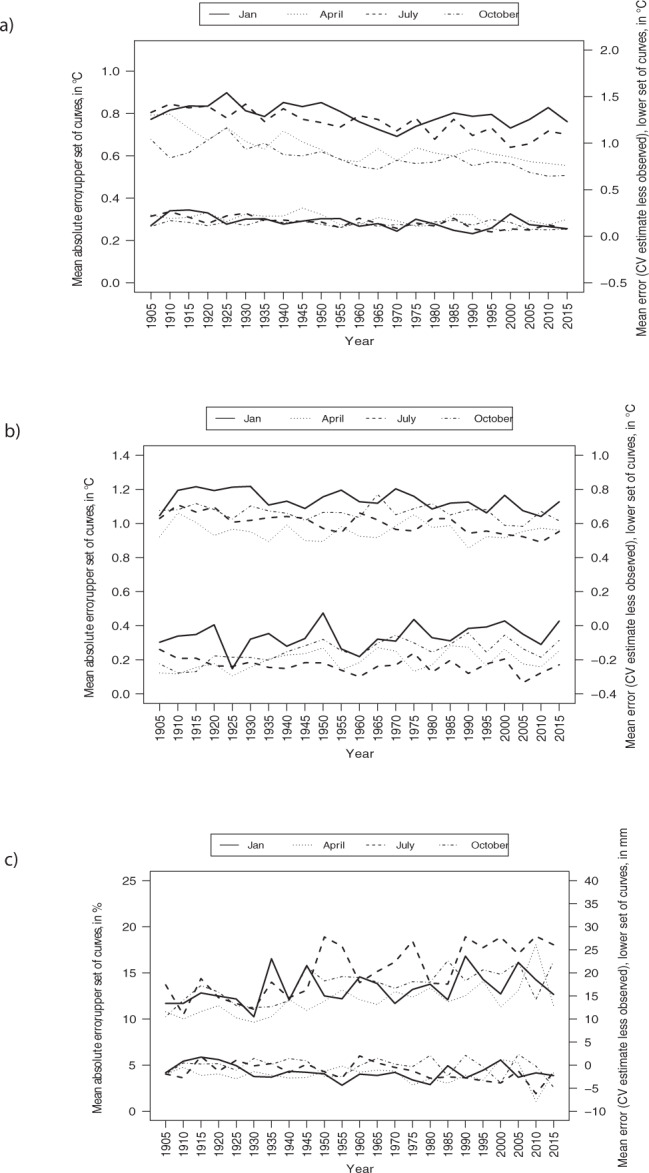
Temporal variation in mean absolute error (left axis: upper set of curves on each graph) and bias (right axis; lower set of curves on each graph) for a set of 160 stations for (**a**) maximum temperature, (**b**) minimum temperature, and (**c**) precipitation. Error rates for January, April, July and October at five year intervals from 1905 to 2015 are plotted separately.

Figure 6 illustrates the MEs (Estimated minus Recorded) and MAEs for January, April, July and October for 160 test stations every five years from 1905 to 2015. The MEs for maximum temperature models ranged between 0.0° and 0.3 °C (Fig. 5a), and from −0.3° to 0.1 °C for minimum temperature (Fig. 5b). The MAEs varied by year from 0.9° to 1.2 °C for minimum temperature, and from 0.5° to 0.9 °C for maximum temperature. MAEs showed a significant declining trend over time for April (maximum temperature), July (minimum temperature, maximum temperature), and October (maximum temperature), as shown in Table 3, suggesting an improvement in the quality of predictions over time. Temperature MEs were not significantly related to year (Table 4).

**Table 3:** Trend in MAEs (January, April, July, and October for the 160 test stations every five years from 1905, 1910…. 2015).

Variable	January	April	July	October
Maximum temperature	−0.026	−0.089*	−0.061*	−0.075*
Minimum temperature	−0.025	−0.006	−0.046*	−0.014
Precipitation	0.015	0.020	0.019	0.015

Values shown are the Pearson correlation coefficient (*r*) indicating the correlation between MAE and year. Starred values indicate statistically significant results at the 0.01 level. Temperature MAEs are expressed in °C and precipitation MAEs are expressed as a percentage of total monthly recorded precipitation.

**Table 4:** Trend in MEs (January, April, July, and October for the 160 test stations every five years from 1905, 1910…. 2015).

Variable	January	April	July	October
Maximum temperature	−0.032	−0.035	−0.042	−0.013
Minimum temperature	−0.025	−0.006	−0.046	−0.014
Precipitation	−0.021	−0.012	−0.043*	−0.014

Values shown are the Pearson correlation coefficient (*r*) indicating the correlation between ME and year. Starred values indicate statistically significant results at the 0.01 level. Temperature MEs are expressed in °C and precipitation MEs are expressed in mm.

For precipitation, the MEs depicted in Fig. 6 ranged between −7.8 mm and 2.4 mm, or 9.7% to 19.0% of the precipitation surface mean. Precipitation MAEs (expressed as a percentage of the recorded value) were not significantly correlated with year (Table 3; see also 10.17605/OSF.IO/2DAK5). Precipitation MEs were not significantly correlated with year (Table 4), with the exception of July MEs which showed a significant declining trend over time. Cross-validation estimates and recorded values for the 160 test stations (every five years from 1905 to 2015) can be obtained from MacDonald^13^.

McKenney *et al*.^4^ assessed the 2006 models using a representative withheld sample of between 100 and 200 stations (increasing over the course of the century), which were not used in the creation of the spatial models. By comparison, the current study compared CV estimates to recorded values for 160 stations selected to be more representative than the full sample. We used a leave-one-out approach as opposed to withholding a set of stations simultaneously. We therefore urge some caution in directly comparing the MEs and MAEs between the two studies due to these methodological differences.

## Usage Notes

The monthly historical spatial models presented in this manuscript will be of interest to researchers and practitioners that need historical estimates of temperature or precipitation variables for points or regions in North America. These temperature and precipitation estimates are central inputs to species richness^27^, plant hardiness^28^, forest productivity^29^, forest cover change^30^, carbon^31,32^, water budget^33^, and species distribution models^34^, as well as in determining representativeness of different locations for conservation research^35^. Further, models based on gauge data are also used as inputs to satellite-based precipitation estimates^36^.

We note there are regional limitations associated with spatial models, especially for precipitation-related variables. Model predictions of precipitation along the coast were associated with larger errors, suggesting that a distance to coast independent variable might improve these estimates. For applications that exclusively include coastal areas of North America, more specialized gridded products may be more appropriate. However we note the paucity of station observations in some regions to develop/calibrate such models is especially problematic in Canada.

## Data Availability

SAS code used for data preparation and analysis has been published at Open Science Framework under the same name as the publication (10.17605/OSF.IO/2DAK5)^37^. SAS code and output for the residual analysis can be accessed from this DOI.
